# Resilience and clinical recovery in first-episode schizophrenia: a 10-year longitudinal study of clinically recovered, non-recovered, and healthy control participants

**DOI:** 10.3389/fpsyt.2026.1813256

**Published:** 2026-10-16

**Authors:** Anne-Kari Torgalsbøen, Christine Mohn, Frank Larøi, Nikolai Czajkowski

**Affiliations:** 1 Department of Psychology University of Oslo, Oslo, Norway; 2 National Centre for Suicide Research and Prevention, Institute of Clinical Medicine, University of Oslo, Oslo, Norway; 3 Psychology and Neuroscience of Cognition Research Unit, University of Liège, Liège, Belgium; 4 Division of Mental Health, Norwegian Institute of Public Health, Oslo, Norway

**Keywords:** first-episode psychosis, high retention, longitudinal study, prognosis, recovery rate, resilience

## Abstract

**Introduction:**

Research on resilience in clinical recovery following first-episode schizophrenia (FES) remains limited, and few longitudinal studies achieve sufficient retention to capture long-term trajectories. To address this, the Oslo Schizophrenia Recovery Study (OSR) was designed to examine resilience over time in FES. Investigating resilience is essential for understanding how individuals exposed to schizophrenia may achieve favorable outcomes. We addressed two primary questions: First, how does resilience change over time in individuals with FES compared with healthy controls. Second, whether longitudinal resilience trajectories within the FES group differ between participants who meet predefined clinical recovery criteria compared to those who do not.

**Methods:**

Repeated assessments were conducted across 12 clinical evaluations. The sample comprised 28 individuals with FES and 28 healthy controls. Resilience was measured with the Connor–Davidson Resilience Scale, and social and role functioning were indexed using the Global Functioning Scales. The data were analyzed using linear multilevel models. Candidate longitudinal mixed-effects models were compared using the small-sample corrected Akaike Information Criterion (AICc), with parameters estimated by maximum likelihood.

**Results:**

At 10-year follow-up, retention was 79%. Half of the FES group achieved clinical recovery. Model estimates indicated increasing resilience over time in the FES group, whereas resilience in healthy controls remained relatively stable, although statistical support for differential trajectories between groups was limited. Clinically recovered individuals showed a steeper increase in resilience. In contrast, non-recovered individuals exhibited only modest, gradual improvements, with resilience levels remaining lower at study completion.

**Conclusion:**

Different resilience trajectories were observed between clinically recovered and non-recovered individuals with FES, with greater improvements in resilience associated with recovery. Although causality remains uncertain and differences to healthy controls were less robust, these findings highlight the value of repeated longitudinal assessments of resilience in FES, both to more precisely characterize resilience trajectories and to reduce drop-out rates.

## 1 Introduction

Resilience science explores the mechanisms of successful adaptation in the face of adversity (1) often described as “ordinary magic” (2). Although there is still no consensus definition, resilience is commonly understood as a dynamic, interactive process spanning biological, psychological, social, and ecological systems that help maintain or enhance mental well-being under risk (3). It involves behaviors, thoughts, and actions that can be developed in anyone and is shaped by environmental context, support systems, coping strategies, and personality-related dispositions, including genetic factors (4–6). The APA Dictionary of Psychology (American Psychological Association) (7) similarly defines resilience as adapting well in the face of adversity, trauma, tragedy, threats, or significant stressors, building on the work of Masten (2) and other key resilience researchers (8, 9). As such, resilience is highly relevant for understanding how individuals with schizophrenia can achieve favorable outcomes despite substantial challenges, even though there is currently no gold-standard measure for assessing resilience in this population (10).

Schizophrenia is characterized by cognitive impairments and pronounced disturbances in self-perception and interpersonal functioning, with substantial consequences for health and overall well-being (11). The illness typically follows a remitting–relapsing course, with considerable variability in outcomes. Recovery rates of up to 57% have been reported among individuals with first-episode schizophrenia (FES) (12).

The increasing recognition that mental health is defined by the presence of positive states and outcomes, rather than merely the absence of disorder (13), has stimulated research on factors that promote recovery in serious mental illness. Despite this, research that specifically examines processes that facilitate recovery remains limited (14). Longitudinal studies have shown a significant relationship between resilience and sustained recovery in older patients with schizophrenia, underscoring resilience as an important factor in maintaining long-term recovery (15, 16).

Recent work in early psychosis has demonstrated lower resilience scores in individuals with first-episode psychosis compared with general population norms (17). Similarly, studies show that individuals at clinical high risk and who later transition to psychosis exhibit reduced resilience, which is associated with more severe negative symptoms and poorer role functioning (18). These findings indicate that diminished resilience is linked to greater psychopathology and less favorable functional outcomes. Consistent with this, in individuals at clinical high risk for psychosis, resilience factors—particularly self-esteem—have been associated with fewer negative and general symptoms and fewer daily hassles, suggesting that enhancing resilience may represent a promising intervention target (19). In schizophrenia spectrum disorders, resilience is reduced compared with healthy controls, and low resilience is associated with poor psychosocial functioning, which improves as resilience increases (20). Despite these studies, longitudinal research on resilience trajectories and their role in clinical recovery in FES remains scarce. To date, the Oslo Schizophrenia Recovery (OSR) study is the only longitudinal study that has tracked resilience in FES, demonstrating different resilience trajectories in clinically recovered compared to non-recovered patients over a 4-year follow-up (21).

In the present study, we extend this work by examining resilience trajectories over a 10-year period in FES patients—both clinically recovered and non-recovered—compared with healthy controls. The OSR study used a repeated-assessment design with annual resilience assessments and a total of 12 clinical evaluations over 10 years, enabling a detailed investigation of resilience in the same individuals over time. Specifically, we addressed two research questions:

1. How does resilience develop over time in individuals with FES compared with matched control participants?
2. Within the FES group, how do longitudinal resilience trajectories differ between participants who meet pre-specified clinical recovery criteria compared to those who do not?

## 2 Methods

### 2.1 Participants

From 2007-2011, 31 participants were referred to the OSR-study from various mental health service institutions in the Vestre Viken Hospital Trust (VVHF) and the Oslo metropolitan area in Norway, the majority coming from units specializing in early intervention and treatment of psychosis. This catchment area consists of rural areas as well as city centers and provides state-funded healthcare to the south-eastern part of Norway. The patients were provided with treatment by their local mental health service institutions. The treatment was given independently of the study, and included psychoeducation and/or cognitive behavior therapy, case management and antipsychotic medication (80% were prescribed second-generation antipsychotics).

To recruit first-episode schizophrenia spectrum patients, the inclusion criteria were well defined. Twenty-eight out of the 31 referred participants fulfilled the following inclusion criteria: age >18 years, the first episode of mental illness was within the spectrum of schizophrenia and psychosis according to DSM-IV (22) and referral occurred within the first five months of their first contact with a hospital or outpatient clinic due to psychosis. Exclusion criteria were diagnosis of an affective disorder, IQ <70, and head trauma. All participants could read and write Norwegian fluently.

### 2.2 Healthy controls

A control group of 28 participants was pairwise matched to patients by gender, age, and education (± 1 year). Younger controls were recruited from junior and senior high schools around Oslo; older controls via electronic ads on the Vestre Viken Hospital Trust (VVHF) website (VVHF serves south-eastern Norway, including rural areas and city centers). Assessments took place at hospitals near Oslo or at the University of Oslo. Exclusion criteria included: history of schizophrenia or other severe mental disorder, IQ < 70, neurological disease, head injury, or loss of consciousness >10 minutes, current psychotropic medication or narcotics for pain, chronic somatic illness causing significant fatigue or pain, alcohol/substance abuse, dyslexia or other major learning difficulties, and insufficient spoken/written Norwegian. After carefully describing the study and the procedures involved, written informed consent was obtained from all participants. The study was approved by the Regional Committee for Research Ethics (REK) (2007/1139) and conducted according to the Helsinki declaration.

Complete data are available for all 12 assessment time points over 10 years for the patient group. They were assessed at baseline, after six months and after a year. Thereafter, they were assessed every year for 9 consecutive years. Twenty-two of the initial 28 were available at the 10-year follow-up, resulting in a retention rate of 79%. The healthy control group was available for resilience assessment at year 4 and 10. All participants in the patient group were retained during the three first follow-up assessment time points. Six participants dropped out of the study: three dropped out during the two-year follow-up, and an additional three participants dropped out during the 3-year follow-up. Dropout reasons were mainly refusal to participate due to anxiety, a lack of insight into having a mental illness, and not finding participation in research useful. One participant did not provide a reason for dropout, and another was untraceable. The only significant baseline difference at the 10-year follow-up was higher scores for non-completers on Positive (p=0.008) and General symptoms (p=0.023) on the Positive and Negative Syndrome Scale (PANSS). The participant flow is shown in **Table 1**.

**Table 1:** Participant flow and resilience assessments across 10 years.

Time point	FES (N)	Controls (N)	Lost to follow-up (FES) (N = 6)	Completed resilience assessment with CD-RISC(N)
Baseline	28			28
6 Months	28			28
Year 1	28			28
Year 2	25		3	25
Year 3	22		3	22
Year 4	22	28		50
Year 5	22			22
Year 6	22			22
Year 7	22			22
Year 8	22			22
Year 9	22			22
Year 10	22	28		50

### 2.3 Assessment and measures

Clinical interviews and resilience assessments were conducted by an experienced clinical psychologist. Diagnoses were made by treating clinicians, then was separately confirmed by the psychologist at study entry using The Structured Clinical Interview for DSM-IV Axis I disorder (SCID-I) (23) and evaluated with the Positive and Negative Syndrome Scale (PANSS) (24). A semi-structured interview specific to the OSR study assessed social and role functioning through the Global Functioning Scales: Social (GFS: Social) and Role (GFS: Role) (25). These 10-point scales effectively differentiate social from work/school functioning, are sensitive to changes in first-episode patients, and appear to be a useful and valid measure of the more subtle characteristic often seen in first-episode patients (26). The Social scale evaluates peer relationships and family involvement, while the Role scale assesses performance in school, work, or homemaking. These measures are appropriate for the prospective monitoring of individuals with FES (27). Follow-up assessments were performed by psychologists trained in PANSS ratings and an inter-rater reliability assessment confirmed satisfactory agreement on remission and recovery judgments (21).

In the FES group, the Connor–Davidson Resilience Scale (CD-RISC) (28) was assessed at each follow-up, while in the healthy control group, CD-RISC data were available only at the 4 and 10-year follow-up. This scale was translated into Norwegian and has demonstrated good psychometric properties (29, 30). The 25 items that comprise the scale are each rated on a 5-point Likert scale (0 = not true at all, 4 = true nearly all the time). Items consist of statements such as “I am able to adapt to change”, “I tend to bounce back after illness or hardship”, and “I can handle unpleasant feelings”. Higher scores reflect high levels of resilience.

Clinical recovery in this study was defined according to the operational criteria suggested by Liberman et al. (31) which is suitable for FES. These criteria consist of: (1) psychotic symptom remission with PANSS scores P1 (delusions), P2 (conceptual disorganization), P3 (hallucinatory behaviors), G5 (mannerisms and posturing), G9 (unusual thought content), N1 (blunted affect), N4 (social and emotional withdrawal), N6 (lack of spontaneity) below 4 (32), and (2) adequate functioning consisting of at least part-time ordinary work (paid) or school, living independently (without supervision by family or other residential support), and socializing with peers and/or dating or otherwise involved in recreational activities that are age-appropriate and independent of professional supervision (Social and Role score 8), and (3) duration of symptom remission and adequate functioning for two years.

### 2.4 Statistical analyses

All statistical analyses were conducted using IBM SPSS version 27 and R version 4.3.1, with the `lme4` package for linear mixed-effects models. Longitudinal growth curve models were used to examine resilience trajectories over 10 years, allowing us to estimate individual change over time while accounting for unobserved heterogeneity. Multilevel modeling (MLM) efficiently handles partially missing data by including all available observations without listwise deletion, which is particularly important given our sample size (33)). Recent work indicates that linear mixed models (LMMs) perform well even in relatively small samples (34), and that they are preferable to traditional methods such as repeated-measures ANOVA.

All mixed models were estimated using maximum likelihood (ML), which yields valid inference under a Missing At Random (MAR) assumption conditional on the variables included in the model. Our main longitudinal models included group (FES vs. control) and time (years since baseline), as well as their interaction. Because baseline symptom severity differed between completers and non-completers in the FES group, we conducted a sensitivity analysis of FES participant in which baseline PANSS was included as covariates. This analysis was conducted to evaluate whether symptom-related attrition influenced the estimated resilience trajectory.

All candidate models were estimated using maximum likelihood, allowing models with different fixed-effect specifications to be compared. Model selection was based on the small-sample corrected Akaike Information Criterion (AICc) (35). AICc applies a penalty for model complexity and reduces the tendency to favor overly complex models when the sample size is modest. Lower AICc values indicate greater relative support among the candidate models.

### 2.5 Longitudinal modeling: FES versus controls

To address the first research question, we conducted a series of linear mixed models to examine resilience trajectories over time across all participants (FES and controls). We began with a null model (random intercept only) and then incrementally added fixed effects for time, random slopes for time, group (FES vs. control), and the time × group interaction.

The fixed effects represented the estimated baseline resilience level in the control group, average yearly change in resilience among controls, the estimated baseline difference between the FES and control groups, and the difference between groups in their rate of change over time. The random effects included individual differences in estimated baseline resilience (random intercept) and change over time (random slope), together with residual within-person variability.

From the best-fitting model, we generated predicted CD-RISC scores and plotted predicted and observed trajectories for descriptive purposes (**Figure 1**). Fixed and random effect estimates for these models are presented in **Table 2**.

**Figure 1:**
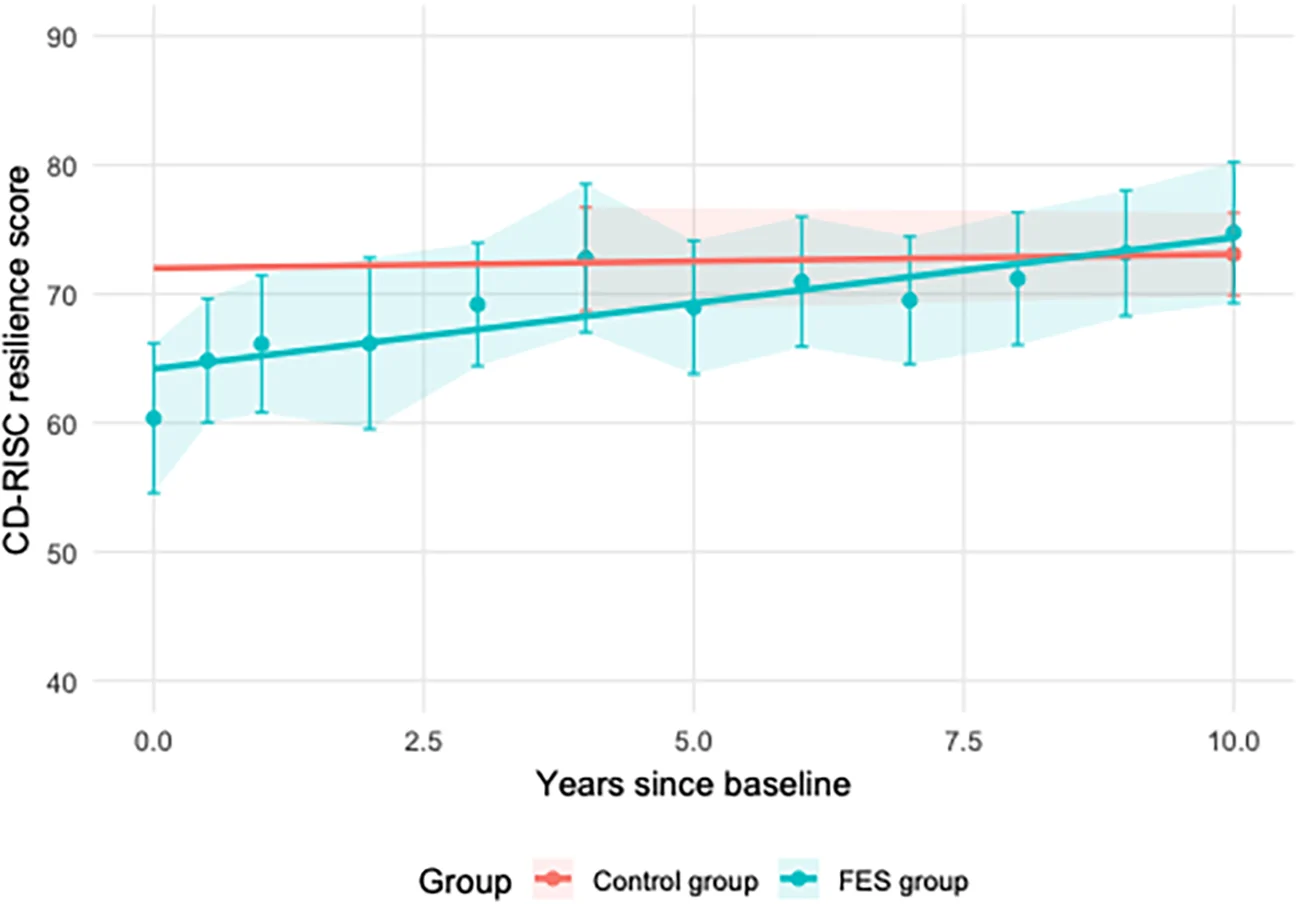
Observed mean resilience and model-predicted longitudinal trajectories in first-episode schizophrenia (FES) and healthy controls. Points show observed mean CD-RISC scores at each available assessment, error bars indicate 95% confidence intervals, and solid lines represent trajectories estimated from the longitudinal mixed-effects model. Mean values of resilience for clinically recovered and non-recovered at each assessment point (see **Supplementary Tables**; **Table 3**).

**Table 2:** Linear mixed-effects growth models of resilience (CD-RISC) over 10 years in FES patients and healthy controls.

Predictors	m1* E**	m2 *E*	m3 *E*	m4 *E*	m5 *E*
(Intercept)	69.91 ^***^	65.10 ^***^	65.62 ^***^	67.13 ^***^	72.00 ^***^
Years		0.93 ^***^	0.85 ^***^	0.81 ^***^	0.11
FES (Control=0, FES =1)				-2.04	-7.81
Years: FES					0.91
Random Effects					
Residual variance	111.35	101.83	89.93	89.76	89.38
Random intercept variance	56.73 _id_	54.05 _id_	81.14 _id_	78.72 _id_	77.04 _id_
Random time variance			0.96 _id.time_	0.98 _id.time_	0.93 _id.time_
AIC	2515.309	2491.501	2483.806	2481.451	2480.201
AICc	2515.384	2491.627	2484.071	2481.805	2480.658

p<0.05   ** p<0.01   *** p<0.001

M*= model.

E**=Estimates.

### 2.6 Longitudinal modeling: recovered versus non-recovered FES patients

To address the second research question, we restricted analyses to the FES group and compared resilience trajectories between clinically recovered and non-recovered patients at the 10-year follow-up. We fitted linear mixed models including main effects of time and recovery status (recovered vs. non-recovered), as well as their interaction. We examined both simple linear time effects and piecewise (segmented) linear models with distinct slopes for predefined time intervals to capture potential non-linear patterns of change.

Visual inspection of the observed trajectories suggested that the rate of change differed between the early and later follow-up periods. Given the modest sample size, we used a piecewise linear specification rather than more flexible non-linear models. The primary piecewise model used a common breakpoint at one year for both recovery groups, yielding separate slopes for the period from baseline to one year and the period from one to ten years. To evaluate whether the findings depended on the precise placement of this breakpoint, sensitivity analyses were conducted using otherwise identical models with alternative breakpoints at six months and two years.

Model selection again followed an information-theoretic approach based on AICc, comparing nested models differing in their time specification (e.g., linear vs. piecewise) and inclusion of the time × recovery interaction. The best-fitting model was used to obtain predicted trajectories which were plotted alongside observed CD-RISC scores (**Figure 2**). Detailed estimates for all candidate models are provided in **Supplementary Table 3**.

**Figure 2:**
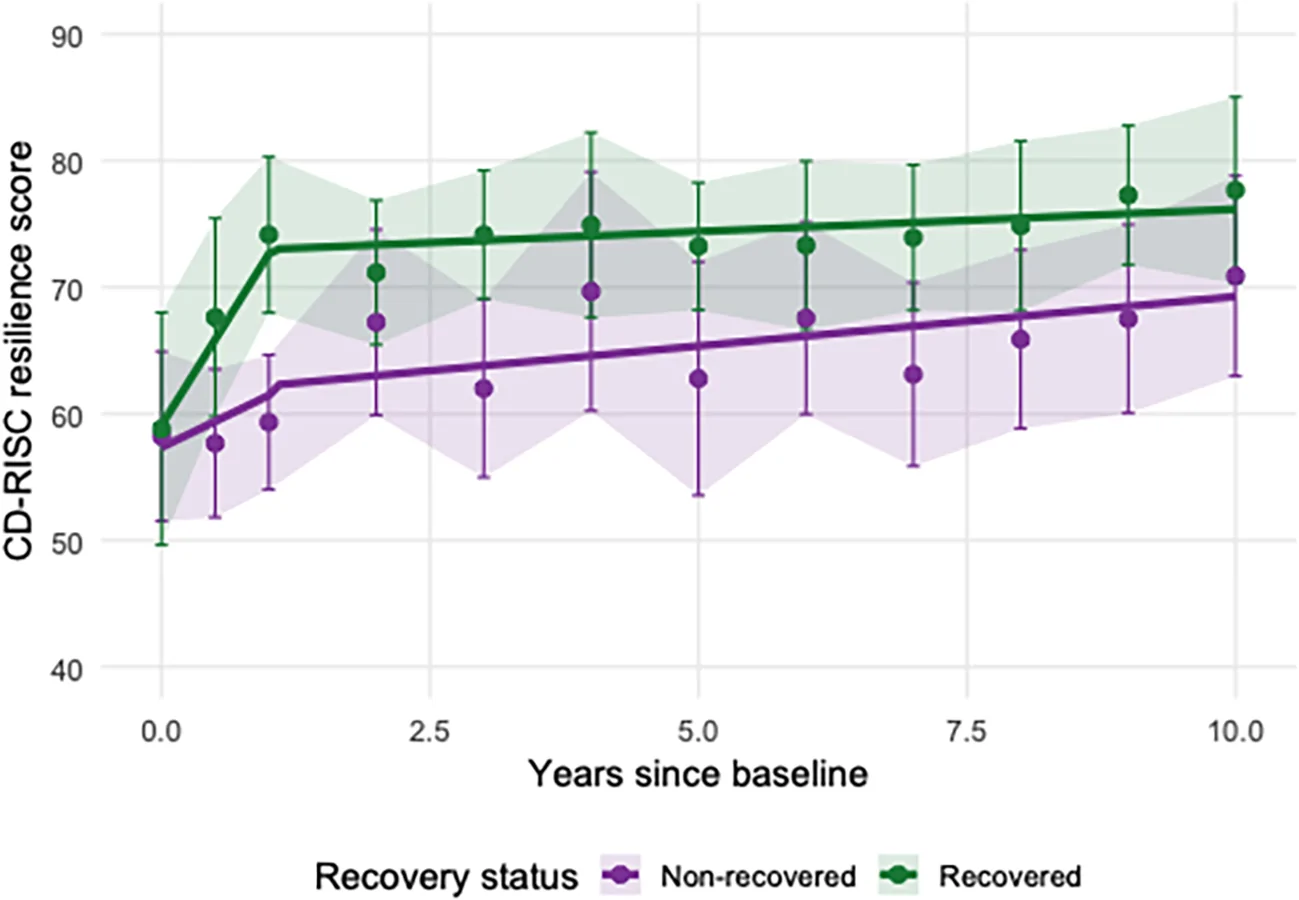
Observed mean resilience and model-predicted longitudinal trajectories in clinically recovered and non-recovered first-episode schizophrenia (FES) patients. Points show observed mean CD-RISC scores at each assessment, error bars indicate 95% confidence intervals, and solid lines represent trajectories estimated from the piecewise longitudinal mixed-effects model.

## 3 Results

### 3.1 Sample characteristics

**Table 3** shows demographic characteristics of the participants at baseline. **Table 4** shows clinical characteristics of the patient group at baseline and at 10-year follow-up. See **Supplementary Table 1** for demographic and clinical characteristics of clinically recovered and non-recovered patients at 10-year follow-up.

**Table 3:** Demographic characteristics of patients and controls at baseline.

	FES (n=28)	Controls (n=28)
Demographics		
Age*	21.0 (2.6)	21.1 (2.7)
Gender		
Women	39.3% (n=11)	39.3% (n=11)
Men	60.7% (n=17)	60.7% (n=17)
Level of education		
Elementary school	39.3% (n=11)	32.1% (n=9)
High school	28.6% (n=8)	57.1% (n=16)
Some college	25.5% (n=7)	7.1% (n=2)
BA or higher**	7.2% (n=2)	3.6% (n= 1)

*Numbers in mean (SD) for age.

**BA, bachelor’s degree.

**Table 4:** Clinical characteristics of FES at baseline and at 10-year follow-up.

	Baseline (n=28)	10 years (n=22)
Diagnosis		
Schizophrenia	21	2
Schizoaffective	6	5
Residual schizophrenia	0	1
Psychosis NOS	1	
Substance use, current	3.6% (n=1)	9.1% (n=2)
Substance use, previous	64.3% (n=18)	
Psychosis symptoms (PANSS)*		
Positive	18.3 (5.4)	8.1 (2.3)
Negative	20.7 (4.3)	8.6 (2.1)
General	40.2 (9.3)	19.9 (3.9)
Global functioning (GAF)		
Social	6.1 (1.2)	7.1 (1.2)
Role	4.1 (1.9)	6.8 (1.4)
Clinical recovery**	None	50% (n=14)
Medication***	92.8% (n=26)	68.2% (n=15)
In current relationship	0	29% (n=8)
In work / education	0	46% (n=13)

*Numbers in mean (SD) for PANSS and GAF.

**The calculation of rate of recovery is based on all the 28 baseline subjects in the study.

***Medication: antipsychotics, mood stabilizing, and/or antidepressants.

### 3.2 Resilience trajectories in FES patients and healthy controls

Resilience trajectories over 10 years for FES patients and healthy controls were investigated in a set of linear mixed-effects models. Model comparisons using the small-sample corrected Akaike Information Criterion (AICc) indicated only modest differences among the candidate models, with a model that included a group-by-time interaction effects (m5) receiving the greatest overall support. However, the small AICc differences indicated considerable model-selection uncertainty (**Table 2**).

Fixed-effect estimates suggested lower model-estimated resilience among FES participants at the start of the modeled trajectory, but this group coefficient was not statistically significant. The estimated time × group interaction suggested greater increases in resilience in the FES group than in controls, but this interaction did not reach statistical significance. Random intercept and slope variances indicated considerable individual variability in both estimated initial resilience and change over time.

On average, resilience increased over time in both groups, with the largest gain observed in the FES group, whereas controls showed comparatively stable mean levels across the 10-year period. Individual trajectories within both groups were heterogeneous, with some participants showing marked increases and others showing relatively flat courses (**Figure 1**). In a sensitivity analysis on data from FES participants, adjustment for baseline PANSS positive and general psychopathology scores did not substantially alter the estimated time effect for resilience.

### 3.3 Resilience trajectories in clinically recovered and non-recovered FES patients

Analyses addressing the second research question were restricted to the FES group and compared resilience trajectories between patients who were clinically recovered and those who were not at the 10-year follow-up.

The best-fitting model (m8r; AICc = 1926.55; **Supplementary Table 3**) was a piecewise linear mixed-effects model including recovery status, with distinct slopes for the period from baseline to one year and the period from one to ten years. In a sensitivity analysis comparing otherwise identical piecewise models with breakpoints at six months, one year, and two years, the one-year breakpoint received the strongest support. The alternative six-month and two-year specifications had AICc values of 3.39 and 7.48, respectively.

In this model, baseline resilience levels did not differ significantly between patients who were later classified as clinically recovered and those who remained non-recovered, indicating comparable starting points. Recovery status, however, was strongly associated with subsequent resilience trajectories: individuals who ultimately achieved clinical recovery showed a markedly steeper increase in resilience during the first year after baseline, followed by more gradual gains thereafter.

In contrast, non-recovered patients exhibited only modest, gradual improvements in resilience across the entire 10-year follow-up, and their mean resilience levels remained clearly below those of the clinically recovered group at study completion. Predicted and observed trajectories from the piecewise model (**Figure 2**) thus indicated a non-linear resilience pattern among patients who recover clinically, characterized by an early, rapid increase and slower later gains, whereas non-recovered patients showed a flatter, more attenuated trajectory over time. Detailed parameter estimates for all candidate models are provided in **Supplementary Table 3**.

## 4 Discussion

Using a prospective design with repeated assessments on a well-defined FES-group and healthy controls with a low dropout rate over 10 years, we found evidence for a differential development of resilience across the groups. This pattern may reflect two possible facets of resilience. In the FES group, the observed increase in resilience over time could possibly be interpreted as a gradual recovery from an initial reduction during the acute phase of illness, consistent with a potential “bouncing back” process, although this interpretation is not directly demonstrated by our data. In contrast, resilience in the control group appeared relatively stable, which might suggest that, in the absence of major adversity, resilience is stable over time and only becomes most apparent when individuals are exposed to significant stressors or environmental threats (8). These interpretations are tentative and should be regarded as possible explanations of the observed trajectories rather than definitive conclusions about underlying mechanisms. Our data do not allow us to establish causality or to confirm the “bouncing back” process, and thus further longitudinal research is needed to test this hypothesis.

In line with resilience theory (2, 3, 8), we view resilience as a dynamic process, not a fixed trait. Because it was first assessed after illness onset, we cannot disentangle premorbid resilience from adaptations to psychosis, treatment, and context. Our results therefore show associations between resilience trajectories and clinical course but do not establish whether premorbid resilience influences recovery.

Based on the fixed-effect estimates from the best fitting mixed model it is noteworthy that the increase in resilience in FES approaches the level of the resilience scores of controls at 10-year follow-up (**Figure 1**; **Table 2**). We have also confirmed that the recovery rates previously reported in the OSR study (21, 36) were mainly sustained for most clinically recovered participants.

Our analyses addressing the second research question showed that clinically recovered and non recovered FES participants (**Figure 2**) follow different resilience trajectories. Clinically recovered individuals exhibited a non linear course with an early, rapid increase in resilience followed by slower gains, consistent with the rapid recovery trajectory described by Bonanno et al. (9). In contrast, non recovered individuals, including those in symptom remission, showed a more gradual and fluctuating improvement across ten years, aligning with Bonanno’s slow recovery trajectory. These results highlight the importance of considering both timing and rate of change when characterizing recovery in FES. Building on the premise that FES patients respond differently to the adversity posed by schizophrenia (37), these different trajectories suggest that rapid resilience gains may be a hallmark of clinical recovery. In non recovered individuals, the more fluctuating pattern may reflect greater vulnerability to environmental stressors. The rapid development of resilience in the clinically recovered group could be influenced by factors such as spontaneous (endogenous) remission, a highly effective response to psychopharmacological treatment, or neural development/maturation in this young cohort, which may reduce the extent to which the illness disrupts premorbid resilience.

Psychological and social factors, such as having intimate relationships, and working could play a role, potentially serving more as sustaining influences over time. At 10 years, 93% of the clinically recovered participants were employed, and 57.2% were in relationships (see **Supplementary Table 1**). Luthar (38) has shown that strong intimate relationships provide both the support and the informal social control that is needed to negotiate ongoing challenges in life. Having an income from a regular job creates independence, which is considered a part of resilience (39). Another aspect of resilient functioning is the ability to quickly recover from negative affective events (40), claiming that resilient individuals experience negative affect in the same way as non-resilient individuals, but that they can recover more quickly and return more easily to a positive affective state. Further, resilient individuals are better able to learn from their experiences of negative emotions than non-resilient individuals. Early treatment, access to vocational and social participation as well as participating in a positively framed research project as the OSR-study, are all psychosocial protective environmental factors that may have contributed to the positive outcome of the participants in the present study. Increased openness and more positive public perceptions in Norway towards mental illness (41) may influence the development of resilience, as the ecological system is a crucial component of this resilience process (3).

Since the greatest improvement among those who are clinically recovered in year 10 seems to take place during the first year of treatment, this represents a window of opportunity for promoting positive adaptation and personalized treatment. Individual resiliency training (42, 43) designed to improve well-being and social functioning through focusing on a patient’s strength, might be a valuable supplement for less resilient patients in reducing their vulnerability for future episodes.

### 4.1 Strengths and limitations

The main strengths of our study include its repeated-measures design, the inclusion of a healthy control group, and a low dropout rate in both groups. This allowed us to analyze all available data using multilevel models, thereby strengthening the robustness of our findings. Our retention rate of 79% preserves internal and external validity (44) and is notably high compared with similar longitudinal studies in FES (45, 46).

The small sample size is an obvious limitation and may reduce the robustness of our findings. Large samples are generally difficult to obtain in longitudinal studies, especially when multiple repeated assessments are required. Although attrition was modest, baseline symptom differences between completers and non-completers indicate that data may not be strictly Missing At Random (MAR), whereas the mixed models rely on an MAR assumption conditional on the variables included in the model. Sensitivity analyses adjusting for baseline PANSS Positive and General Psychopathology scores did not substantially alter the estimated resilience trajectories. Nevertheless, some bias due to non-random attrition cannot be ruled out.

Further limitations include the absence of conclusive evidence for causal relationships and a likely higher prevalence of comorbid conditions in the patient group, both of which may have influenced the findings. A related limitation is that resilience was not assessed prior to the first psychotic episode.

Resilience was measured with a self-report scale and not corroborated by clinician-rated measures. Self-report may be affected by insight, illness stage, symptom severity, and cognitive or functional impairments in FES. In addition, favorable outcomes among individuals with sustained clinical recovery may have shaped their attitudes, potentially inflating resilience scores.

In conclusion, our findings suggest that resilience develops differently in clinically recovered versus non-recovered individuals with FES, with greater improvements in resilience accompanying clinical recovery. Although causal relationships cannot be established and differences from healthy controls were less robust, our results highlight the value of repeated longitudinal assessments of resilience in FES, both to more precisely characterize resilience trajectories and to help reduce drop-out.

## Data Availability

The raw data supporting the conclusions of this article will be made available by the authors, without undue reservation.
