# Supplementary material for: Resilience and clinical recovery in first-episode schizophrenia: a 10-year longitudinal study of clinically recovered, non-recovered, and healthy control participants
Source: Front Psychiatry. 2026 Oct 16;17:1813256. doi: 10.3389/fpsyt.2026.1813256 (PMC13719347; doi:10.3389/fpsyt.2026.1813256)
Supplement: Supplementary file 1 [file Supplementaryfile1.docx]

**Supplementary tables and figures.**

*Corresponding author Anne-Kari Torgalsbøen: a.k.torgalsboen@psykologi.uio.no*

*Department of Psychology, University of Oslo, PO Box 1094, 0317 Oslo, Norway,*

*telephone 47 47259465.*

**Table S1. Demographic and clinical characteristics of clinically recovered and non-recovered at 10-year follow-up (N = 22).**

|  | **Clinically recovered (n = 14)** | **Not recovered (n = 8)** |
| --- | --- | --- |
| **Age in years** | 32.0 (2.8) | 31.0 (2.6) |
| **Sex**  Female  Male | 57.2 % (n = 8)  42.9 % (n = 6) | 25.0 % (n = 2)  75.5 % (n = 6) |
| **In work/in education** | 92.9 % (n = 13) | 25.0 % (n = 2) |
| **Civil status**  In a relationship  Single | 57.2 % (n = 8)  42.9 % (n = 6) | 100 % (n = 8) |
| **Treatment status**  Not in treatment  Outpatient  Other | 64.3 % (n = 9)  35.7 % (n = 5) | -  75.0 % (n = 6)  25.0 % (n = 2) |
| **Substance abuse** |  | 25.0 % (n = 2) |
| **On antipsychotic medication** | 50.0 % (n = 7) | 83.5 % (n= 7) |
| **In remission** | - | 27.2 % (n=6) |
| **Not in remission** | - | 9.0 % (n=2) |

**Table S2. Mean values of resilience for clinically recovered and non-recovered at each assessment point.**

|  | Non-recovered at year 10 | Clinically recovered  at year 10 |
| --- | --- | --- |
| 0.0 | 58.22222 | 58.81818 |
| 0.5 | 57.66667 | 67.61538 |
| 1.0 | 59.33333 | 74.15385 |
| 2.0 | 67.22222 | 71.15385 |
| 3.0 | 62.00000 | 74.15385 |
| 4.0 | 69.66667 | 74.92308 |
| 5.0 | 62.77778 | 73.23077 |
| 6.0 | 67.55556 | 73.30769 |
| 7.0 | 63.11111 | 73.92308 |
| 8.0 | 65.88889 | 74.84615 |
| 9.0 | 67.50000 | 77.27273 |
| 10.0 | 70.88889 | 77.66667 |

**Table S3. Multilevel models of resilience trajectories in FES patients over 10 years.**

|  | **m1r** | **m2r** | **m3r** | **m4r** | **m5r** | **m6r** | **m7r** | **m8r** |
| --- | --- | --- | --- | --- | --- | --- | --- | --- |
| *Predictors* | *Estimates* | *Estimates* | *Estimates* | *Estimates* | *Estimates* | *Estimates* | *Estimates* | *Estimates* |
| (Intercept) | 69.24 ^***^ | 64.42 ^***^ | 64.39 ^***^ | 59.64 ^***^ | 59.81 ^***^ | 58.92 ^***^ | 58.92 ^***^ | 57.35 ^***^ |
| Time (linear effect) |  | 1.05 ^***^ | 1.06 ^***^ | 1.05 ^***^ | 1.00 ^*^ | 1.69 | 1.69 |  |
| Recovered (10 years) |  |  |  | 8.10 ^**^ | 7.80 ^*^ | 6.56 | 6.49 | 1.74 |
| Time × Recovered (10 years) |  |  |  |  | 0.08 | 1.01 | 1.04 |  |
| Time² (quadratic effect) |  |  |  |  |  | -0.07 | -0.07 |  |
| Recovered (10 years) × Time ) |  |  |  |  |  | -0.10 | -0.10 |  |
| Time 1 (0–1 year) |  |  |  |  |  |  |  | 4.10 |
| Time 2 (>1 years) |  |  |  |  |  |  |  | 0.78 |
| Time 1 × Recovered (10 years) |  |  |  |  |  |  |  | 9.44 |
| Time 2 × Recovered (10 years) |  |  |  |  |  |  |  | -0.43 |
| **Random Effects** | | | | | | | | |
| Residual variance | 108.13 | 95.38 | 80.51 | 80.57 | 80.57 | 74.22 | 78.65 | 64.64 |
| Random intercept variance | 52.54 _id_ | 54.98 _id_ | 66.51 _id_ | 51.25 _id_ | 51.26 _id_ | 71.85 _id_ | 53.59 _id_ | 79.60 _id_ |
| Random time variance |  |  | 1.29 _id.time_ | 1.28 _id.time_ | 1.28 _id.time_ | 5.77 _id.time_ | 1.32 _id.time_ | 103.10 _id.time1_ |
|  |  |  |  |  |  | 0.03 _id.time_sq_ |  | 1.58 _id.time2_ |
|  |  |  |  |  |  |  |  |  |
|  |  |  |  |  |  |  |  |  |
|  |  |  |  |  |  |  |  |  |
| AICc | 1988.39 | 1961.31 | 1948.65 | 1944.18 | 1946.29 | 1949.28 | 1945.93 | 1926.55 |
| - *p<0.05   ** p<0.01   *** p<0.001* | | | | | | | | |

m=model

**Figure S1. Individual and model-predicted resilience trajectories by 10-year recovery status.**

This plot illustrates individual and model-predicted resilience trajectories over time for participants stratified by recovery status at the 10-year follow-up. Each colored line represents an individual's resilience trajectory, while the bold black line represents model predictions of resilience. The left panel shows trajectories for individuals who were not recovered at the 10-year follow-up, and the right panel displays trajectories for those who were clinically recovered.
